# Aucubin suppresses TLR4/NF‐κB signalling to shift macrophages toward M2 phenotype in glucocorticoid‐associated osteonecrosis of the femoral head

**DOI:** 10.1111/jcmm.18583

**Published:** 2024-08-09

**Authors:** Chen Yue, Guofeng Cui, Yan Cheng, Xue Zhang, Hong‐feng Sheng, Yidan Yang, Jiayi Guo, Youwen Liu, Bin Xu

**Affiliations:** ^1^ Department of Orthopedics Luoyang Orthopedic Hospital of Henan Province, Orthopedic Hospital of Henan Province Luoyang Henan China; ^2^ Department of Orthopedics Luoyang Central Hospital Affiliated to Zhengzhou University Luoyang Henan China; ^3^ Department of Orthopedics Tongde Hospital of Zhejiang Province Hangzhou Zhejiang China

**Keywords:** Aucubin, femur head necrosis, macrophages, NF‐kappa B, toll‐like receptor 4

## Abstract

In this study, we investigated whether the ability of aucubin to mitigate the pathology of GONFH involves suppression of TLR4/NF‐κB signalling and promotion of macrophage polarization to an M2 phenotype. In necrotic bone tissues from GONFH patients, we compared levels of pro‐inflammatory M1 macrophages and anti‐inflammatory M2 macrophages as well as levels of TLR4/NF‐κB signalling. In a rat model of GONFH, we examined the effects of aucubin on these parameters. We further explored its mechanism of action in a cell culture model of M1 macrophages. Necrotic bone tissues from GONFH patients contained a significantly increased macrophage M1/M2 ratio, and higher levels of TLR4, MYD88 and NF‐κB p65 than bone tissues from patients with hip osteoarthritis. Treating GONFH rats with aucubin mitigated bone necrosis and demineralization as well as destruction of trabecular bone and marrow in a dose‐dependent manner, based on micro‐computed tomography. These therapeutic effects were associated with a decrease in the overall number of macrophages, decrease in the proportion of M1 macrophages, increase in the proportion of M2 macrophages, and downregulation of TLR4, MYD88 and NF‐κB p65. These effects in vivo were confirmed by treating cultures of M1 macrophage‐like cells with aucubin. Aucubin mitigates bone pathology in GONFH by suppressing TLR4/NF‐κB signalling to shift macrophages from a pro‐ to anti‐inflammatory phenotype.

## 1 INTRODUCTION

Glucocorticoid‐associated osteonecrosis of the femoral head (GONFH), a common orthopaedic disease, can cause long‐lasting disability and severely reduce quality of life.^1^ The condition involves osteocyte necrosis, trabecular bone fracture and articular surface collapse, which may lead to hip joint pain and substantial loss of hip joint function.^2^ In Asia, aucubin (also called eucommia glucoside) from the small tree *Eucommia ulmoides* and the shrub *Aucuba japonica* ^3^, ^4^ has been used for millennia against orthopaedic diseases.^5^, ^6^ We previously showed that aucubin can mitigate osteoblast apoptosis in a cell culture model of GONFH,^7^ but its mechanism of therapeutic action remains unknown.

Here we investigated the mechanism by focusing on regulation of macrophage polarization. During GONFH, macrophages infiltrate the necrotic zone of the femoral head,^8^, ^9^, ^10^ where they adopt a pro‐inflammatory M1 phenotype and secrete inflammatory cytokines such as tumour necrosis factor (TNF)‐α and interleukins (ILs) 1 and 6.^11^, ^12^, ^13^ Under normal conditions, M1 macrophages are reprogrammed to adopt an anti‐inflammatory M2 phenotype and secrete anti‐inflammatory cytokines such as transforming growth factor (TGF)‐β and IL‐10, which promote tissue healing and bone reconstruction.^9^, ^14^, ^15^ However, this ‘turning off’ of inflammatory responses appears to be compromised in GONFH.^9^, ^16^ We wondered whether aucubin might exert its therapeutic effects by promoting the shift in macrophage polarization from the M1 to M2 phenotype.

We also wanted to explore which upstream signalling pathways might drive such a polarization shift. We focused on the pathway mediated by Toll‐like receptor (TLR) 4, which has been shown to drive femoral head osteonecrosis.^17^, ^18^ TLR4 signalling, in turn, activates pathways involving the signalling mediator protein myeloid differentiation primary response 88 (MYD88) and the transcription factor nuclear factor κB (NF‐κB).^19^, ^20^ Aucubin has been shown to inhibit TLR4/NF‐κB signalling in hepatocyte and thereby downregulate inflammatory TNF‐α and IL‐1β.^13^

Here we asked whether the ability of aucubin to mitigate the pathology of GONFH involves suppression of TLR4/NF‐κB signalling and promotion of macrophage polarization to an M2 phenotype.

## 2 MATERIALS AND METHODS

### 2.1 Clinical samples

With the approval of the Ethics Committee at the Luoyang Orthopaedic‐Traumatological Hospital of Henan Province (KY2021‐007‐01), we biopsied femoral heads from 20 patients with GONFH or hip osteoarthritis who underwent total hip arthroplasty at the hospital between May 2022 and April 2023. Patients provided written informed consent for the removal and analysis of their tissue samples.

### 2.2 Analysis of clinical samples

Femoral heads were cut into coronal sections, fixed for 1 week in 10% neutral formalin, decalcified for 4–6 weeks in 0.5 M EDTA, embedded in paraffin and sectioned to a thickness of 5 μm. The sections were dehydrated several times in xylene and ethanol, then stained with haematoxylin–eosin as described.^21^ Sections were analysed under a Slideview VS200 microscope (Olympus, Feasterville, PA, USA), and the severity of osteonecrosis was assessed based on the existence of empty lacunae, amorphous substance and trabecular fractures. The inflammatory cells were counted at 100× magnification under the light microscope within three predetermined fields including the right, centre and left of the necrotic area and the mean was calculated as the final result.

Expression in femoral heads of the ‘cluster of differentiation’ proteins CD68, CD80 and CD206 as well as TLR4, MYD88 and NF‐κB p65 was assessed as described.^9^ Formalin‐fixed, paraffin‐embedded sections were deparaffinized, rehydrated, subjected to antigen retrieval and blocking, then immunostained cyclically with the primary antibody: anti‐CD68 antibody (1:200 dilution, Cell Signalling Technology #76437, USA), anti‐CD80 antibody (1:100 dilution, Abclonal #A16039, China), anti‐CD206 antibody (1:100 dilution, Cell Signalling Technology #24595S, USA), anti‐TLR4 antibody (1:100 dilution, Proteintech, #66350‐1‐Ig, China), anti‐MYD88 antibody (1:100 dilution, Affinity #AF5195, China), anti‐NF‐κB p65 antibody (1:100 dilution, Cell Signalling Technology #8242S, USA), followed by horseradish peroxidase‐conjugated secondary antibody using a commercial kit (Akoya Bioscience #NEL861001KT, USA). Subsequently, sections were treated to amplify tyramine signal; they were stripped of bound antibodies and subjected to antigen retrieval. Finally, sections were counterstained with 4,6‐diamino‐2‐phenylindole (DAPI; Beyotime #C1002, China) to label nuclei and imaged under a Vectra Polaris microscope within the Mantra quantitative pathology imaging system (PerkinElmer, Waltham, MA, USA).

Staining intensity due to primary antibodies was quantitated with the aid of an in‐house machine learning algorithm. Cells were defined based on DAPI labelling of the nucleus and immunostaining against macrophage markers. Two investigators independently counted M1 and M2 macrophages in a single field of view at 20× magnification for each section. The mean was calculated as the final result.

### 2.3 Rat model of GONFH

Animal experiments were approved by the Ethics Committee at Luoyang Orthopaedic‐Traumatological Hospital of Henan Province (KY2021‐007‐01). Sprague–Dawley rats 6–10 weeks old weighing 200–400 g (Dossy Experimental Animals Co., Ltd, Chengdu, China) with ad libitum access to food and water were randomised into four groups of 20 animals each. According to previous study,^22^ the rat model of GONFH was set: the GONFH group was injected intramuscularly once on days 1 and 8 with lipopolysaccharide (Sigma‐Aldrich #L4524, USA) at 10 μg per kg, as well as injected intramuscularly with methylprednisolone (Pfizer, Manufacturing Belgium NV) at 25 mg per kg once daily on days 2–4 and 9–11. Disease was induced in the same way in the GONFH‐AU50 and GONFH‐AU100 groups, which subsequently received aucubin (Biorbyt #orb593769, China) at 50 or 100 mg per kg, respectively, in normal saline by gavage from day 1 for 8 weeks. Rats in the control group were injected with normal saline at times when the other groups were injected with lipopolysaccharide, methylprednisolone or aucubin.

### 2.4 Analysis of rat tissues

Femoral heads were carefully isolated, soaked overnight in 4% paraformaldehyde and analysed along the sagittal plane using a micro‐computed tomography scanner (SkyScan1178, Kontich, Belgium) at a resolution of 9 μm per pixel. Images were reconstructed using DataViewer software (Kontich), and bone mass in the femoral head was assessed in terms of local bone mineral density (BMD), fraction of bone volume to total volume (BV/TV), trabecular count (TB. N), trabecular thickness (TB. Th) and trabecular separation (TB. Sp).

Femoral heads were decalcified, sectioned and stained with haematoxylin–eosin (H&E) as described above for clinical samples. Femoral heads were subjected to cyclic immunostaining as described above for clinical samples to assess the expression in femoral heads of F4/80 (antibody: 1:400 dilution, Proteintech, #29414‐1‐AP, China), CD80 and CD206 as well as TLR4, MYD88 and NF‐κB p65. The primary antibody of CD80, CD206, TLR4, MYD88 and NF‐κB p65 were same with those in clinical samples test.

### 2.5 Cell culture model of M1 macrophages

Human THP‐1 monocytes (Procell #CL‐0233, China) were cultured at 37°C in RPMI 1640 (Gibco #11875093, USA) supplemented with 10% foetal bovine serum (FBS; Gibco #10099141C, USA) and 100 U/mL penicillin‐100 μg/mL streptomycin solution (Cytiva #SV30010, USA) as described.^23^, ^24^ The monocytes were subcultured into 6‐well plates and exposed for 24 h to 100 ng/mL phorbol‐12‐myristate‐13‐acetate (PMA; Meilunbio #MB5349‐1, China) in order to induce their differentiation into macrophage‐like cells. These cells were polarised to an M1‐like phenotype by treating them for 24 h with 50 ng/mL lipopolysaccharide (Sigma‐Aldrich #L4524, USA) and 20 ng/mL IFN‐γ (sigma‐aldrich #SRP3058, USA).

M1‐like macrophages were seeded into 96‐well plates (5 × 10^3^ cells per well) and treated 24 h (37°C, 5% CO_2_) with 50, 100 or 200 ng/mL aucubin. Viability was assessed using the Cell Counting Kit‐8 (CCK‐8; Beyotime Biotechnology, # C0038, China). CCK‐8 (10 μL) was added to each well and incubated for 2 h at 37°C in the dark. The absorbance at 450 nm was measured using a microplate reader (Thermo Fisher Scientific, Waltham, MA, USA). Viability was calculated according to the formula:

$$\begin{array}{c} \;\text{Cell viability}\;\left(\%\right)\\ =\left({\mathrm{OD}}_{\text{experimental}}-{\mathrm{OD}}_{\text{blank}}\right)/\left({\mathrm{OD}}_{\text{control}}-{\mathrm{OD}}_{\text{blank}}\right)\times 100\%. \end{array}$$

These experiments served to determine the concentration of aucubin to be used in the experiments described below.

### 2.6 Analysis of macrophage cultures treated with aucubin

M1 macrophage‐like cells in 6‐well plates (initially 1 × 10^5^ per well) were transfected with short interfering RNA targeting TLR4 (5′‐GGACCUCUCUAAGUGUCAATT‐3′) using lentiviruses (GenePharma, Shanghai, China), then treated in triplicate for 72 h with 100 ng/mL aucubin. Cells were collected, washed three times with phosphate‐buffered saline (PBS) and immunostained in the dark for 30 min with allophycocyanin‐conjugated CD80 (BD Biosciences, #564160, USA) to label M1‐like cells and phycoerythrin‐conjugated CD206 (Biolegend #321110, USA) to label M2‐like cells. Then the labelled cells were analysed on a FACSCalibur flow cytometer (BD Biosciences, Franklin Lakes, NJ, USA). In all cases, flow cytometric data were analysed using FlowJo 10.6.2 software (FlowJO LLC, Ashland, OR, USA).

In other experiments, cells were harvested and total RNA was extracted using TRIzol (Invitrogen #12183555, USA) according to the manufacturer's protocol. The RNA was reverse‐transcribed using the Revert Aid RT Reverse Transcription Kit (Thermo Scientific #K1691, USA), and levels of expression of TLR4, MYD88, NF‐κB p65 were quantitated with real‐time PCR using the SYBR Green PCR Master Mix (Thermo Scientific #4367659, USA), specific primers (Table S1) and the 7900 HT Sequence Detection System (Applied Biosystems, USA). Expression levels were determined using the $2^{-\Delta \Delta C_{t}}$ method.

In a third set of experiments, levels of key proteins in treated cells were measured by western blotting as described.^25^ Cells were lysed in RIPA buffer (Beyotime Biotechnology, #P0013C, China), total protein concentration was measured using a BCA assay kit (Cell Signalling Technology #7780S, USA), equal amounts of protein were fractionated using sodium dodecyl sulphate–polyacrylamide gel electrophoresis, and the proteins were transferred onto a polyvinylidene difluoride membrane. The membrane was incubated overnight at 4°C with antibodies against glyceraldehyde 3‐phosphate dehydrogenase (GAPDH; 1:1000 dilution, Cell Signalling Technology #2118S, USA), TLR4 (1:1000 dilution, Proteintech, #66350‐1‐Ig, China), MYD88 (1:500 dilution, Affinity, #AF5195, China), NF‐κB p65 (1:1000 dilution, Cell Signalling Technology #8242S, USA). Next, the membrane was incubated at room temperature for 2 h with an appropriate secondary antibody. Antibody binding was visualised using enhanced chemiluminescence kit (ECL; Santa Cruz #SC‐2048, USA). and quantitated using ImageJ 1.8.0 software (National institutes of Health, Bethesda, MD, USA). Levels of the proteins were normalised to the level of GAPDH.

In a fourth set of experiments, levels of TNF‐α, IL‐1β and IL‐6 secreted by macrophage‐like cells were measured by assaying the culture medium with a commercial enzyme‐linked immunosorbent assay (Elisa) kit (NeoBioscience TNF‐α: #EHC103a.96; IL‐1β: #EHC002b.48; IL‐6: EHC007, China) following the manufacturer's instructions.

### 2.7 Statistical analysis

Data were analysed statistically using GraphPad Prism 8.0 (San Diego, CA, USA). Data were reported as mean ± standard deviation. Differences between two groups were assessed for one‐way analysis of variance, followed by Tukey's post hoc test. Results were considered statistically significant if they were associated with *p* < 0.01 (marked with **) or *p* < 0.05 (marked with *).

## 3 RESULTS

### 3.1 GONFH involves activation of TLR‐4/NF‐κB signalling and macrophage polarization to the M1 phenotype

**FIGURE 1:**
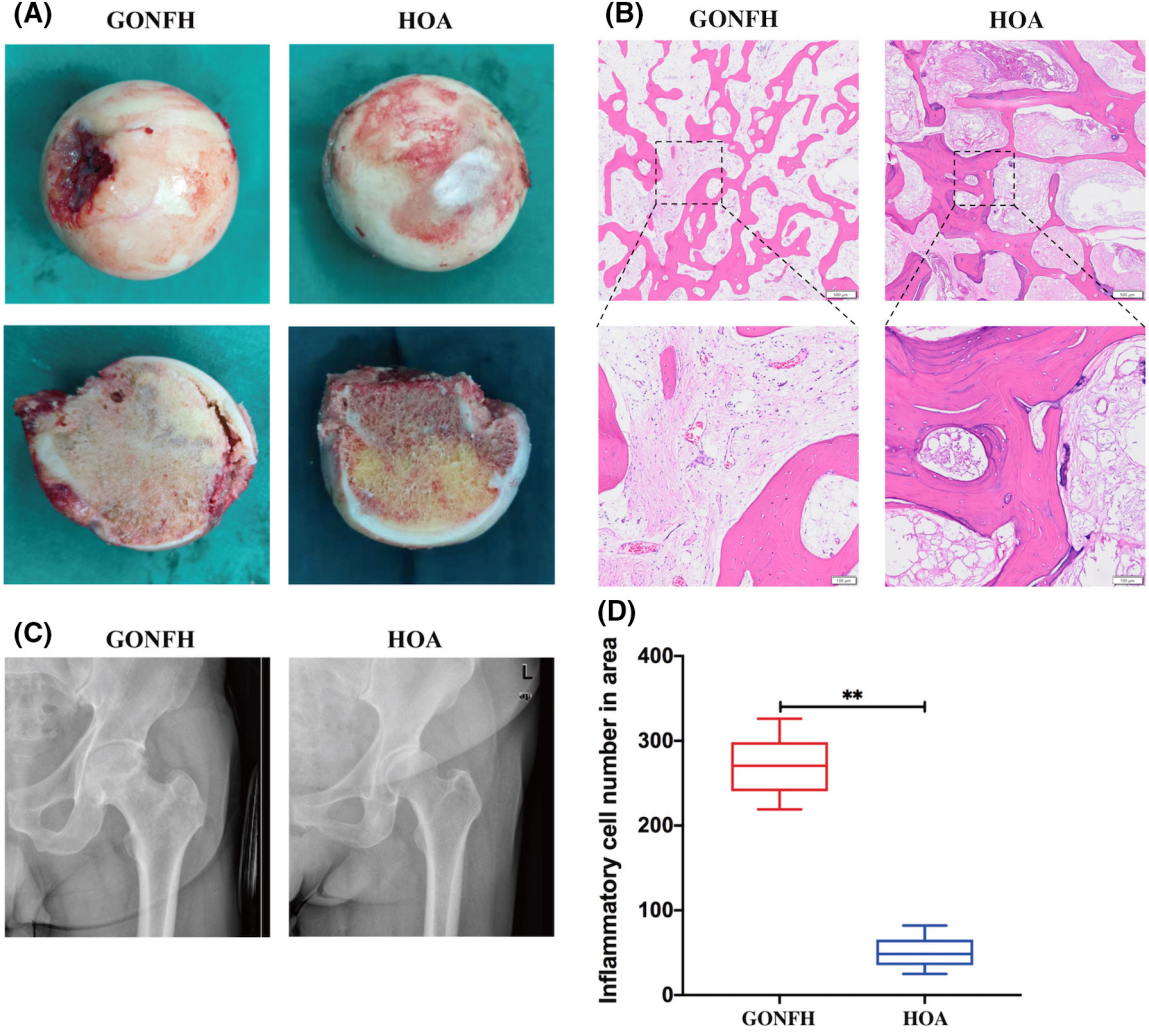
Comparison of femoral head pathology between patients with glucocorticoid‐associated osteonecrosis of the femoral head (GONFH) or hip osteoarthritis (HOA). (A) Representative photographs of femoral heads. (B) Representative photomicrographs of thin sections of femoral head after staining with haematoxylin–eosin. The boxed regions on the top are shown at higher magnification on the bottom. Scale bar on the top, 500 μm. Scale bar on the bottom, 100 μm. (C) Representative X‐ray images. (D) Comparison of infiltration of femoral head tissue by inflammatory cells based on analysis of stained sections like those in panel B. Results are shown for 10 patients per condition (**p < 0.01).

According to the photographs (Figure 1A) and X‐ray images (Figure 1C) of femoral heads, subchondral fractures were observed in GONFH group. While femoral heads from patients with hip osteoarthritis showed lateral joint space narrowing and subchondral sclerosis of the acetabular roof. According to haematoxylin–eosin staining (Figure 1B,D), femoral heads from patients with hip osteoarthritis showed well‐structured trabeculae and minimal infiltration of the inter‐trabecular space by inflammatory cells. In contrast, femoral heads from patients with GONFH showed extensive destruction of bone trabeculae and substantial infiltration of the necrotic area by inflammatory cells.

**FIGURE 2:**
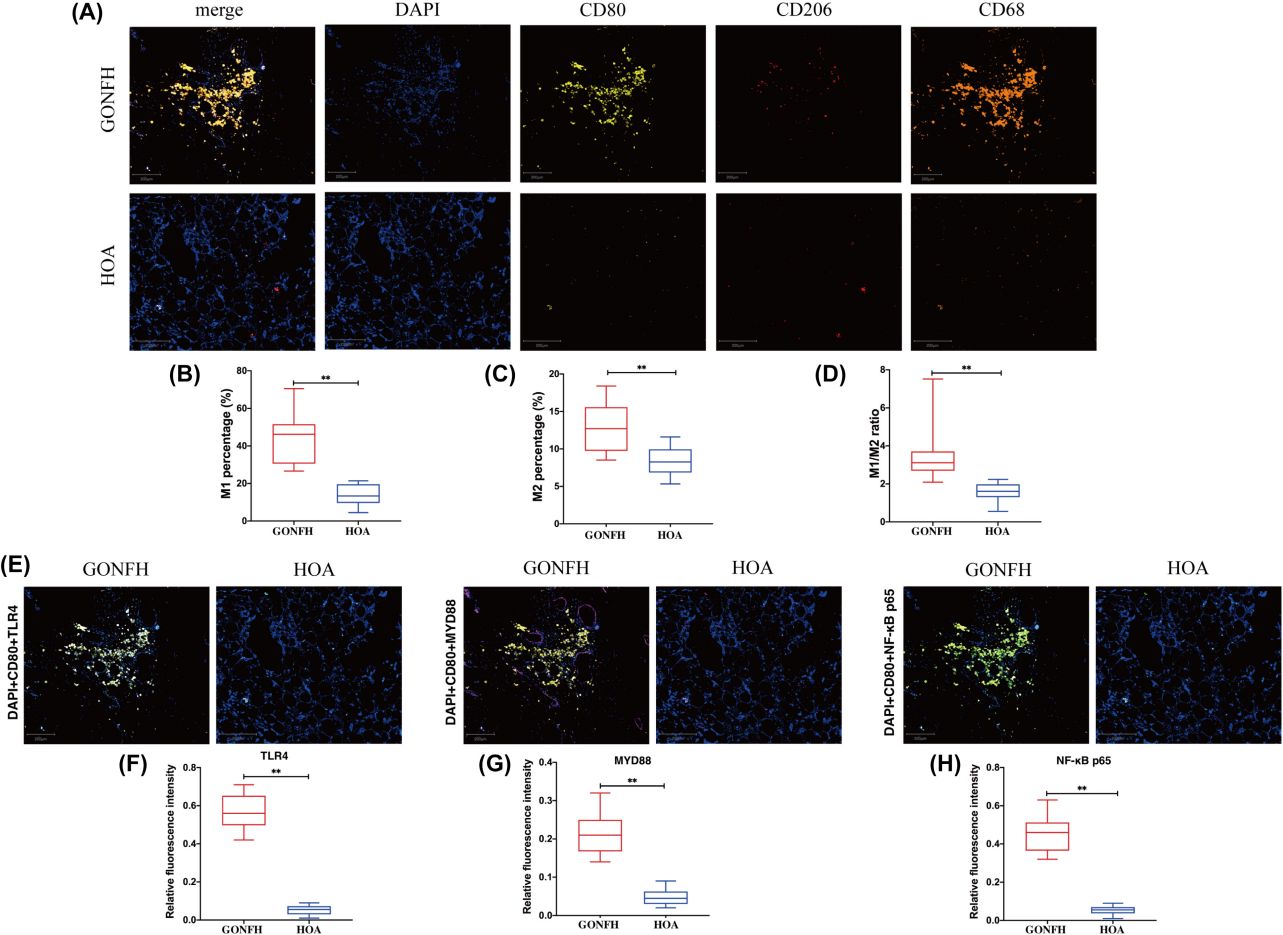
Greater macrophage polarization toward the M1 phenotype and activation of TLR4/NF‐κB signalling in femoral heads of patients with glucocorticoid‐associated osteonecrosis of the femoral head (GONFH) than femoral of patients with hip osteoarthritis (HOA). (A) Micrographs of thin sections of femoral heads after multiplex immunostaining. Scale bar, 200 μm. (B–D) Quantitation of proportions of all macrophages that were polarized to the M1 or M2 phenotype in micrographs like those in panel A. Results are shown for 10 animals per condition (**p < 0.01). (E) Micrographs of thin sections of femoral head after multiplex immunostaining. Scale bar, 200 μm. (F–H) Quantitation of expression of TLR4, MYD44 and NF‐κB p65 in micrographs like those in panel E. Results are shown for 10 patients per condition (**p < 0.01).

Based on multiplex immunohistochemistry, femoral heads from GONFH patients, but not from patients with hip osteoarthritis, showed greater abundance of M1 macrophages (identified based on expression of CD68 and CD80 but no expression of CD206) at the necrotic area and greater abundance of M2 macrophages (identified based on expression of CD68 and CD206 but no expression of CD80). The macrophage M1/M2 ratio was significantly increased in bone tissues samples from GONFH than samples from osteoarthritis (Figure 2). Meanwhile, those samples from GONFH also showed higher expression of TLR4, MYD88 and NF‐κB p65 (Figure 2).

### 3.2 Aucubin ameliorates GONFH pathology in a rat model

**FIGURE 3:**
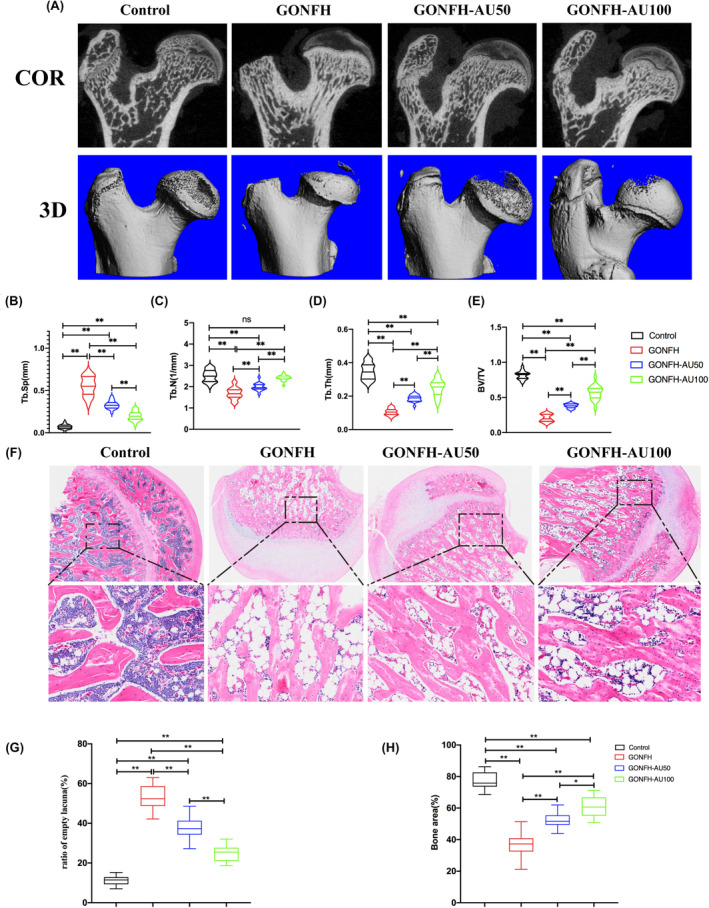
Aucubin ameliorates pathology of glucocorticoid‐associated osteonecrosis of the femoral head (GONFH) in a rat model. Aminals were treated with nothing (‘Control’), injections to induce GONFH (‘GONFH’), or injections to induce GONFH followed by treatment with aucubin at 50 ng/mL (‘GONFH‐AU50’) or 100 ng/mL (‘GONFH‐AU100’). (A) Coronal sections (COR) and 3D reconstructions of the femoral head. (B–E) Micro‐computed tomography to measure trabecular separation (Tb.Sp), trabecular number (Tb.N), trabecular thickness (Tb.Th) and ratio of bone volume to tissue volume (BV/TV). Results are shown for 20 animals per condition. (*p < 0.05, **p < 0.01). (F) Micrographs of thin sections of femoral head after staining with haematoxylin and eosin. The boxed regions on the top are shown at higher magnification on the bottom. Scale bars: Top row, 200 μm; bottom row, 50 μm. (G) Quantitation of the ratio of empty lacuna area to total section area. (H) Quantitation of bone trabecular area. Results are shown for 20 animals per condition. (*p < 0.05, **p < 0.01).

Micro‐CT analysis revealed pronounced bone mineral loss and increased cystic degeneration in the subchondral region of the femoral head in the GONFH group when compared to normal rats (Figure 3A). Tb.Th, BV/TV and Tb. N exhibited significant reductions in the GONFH group accompanied by an increase in Tb. Sp. In contrast to the GONFH group, aucubin could ameliorate the bone necrosis, improve Tb.Th, BV/TV and Tb. N, reduce Tb. Sp. In addition, 100 mg/kg aucubin had better effect than 50 mg/kg.

**FIGURE 4:**
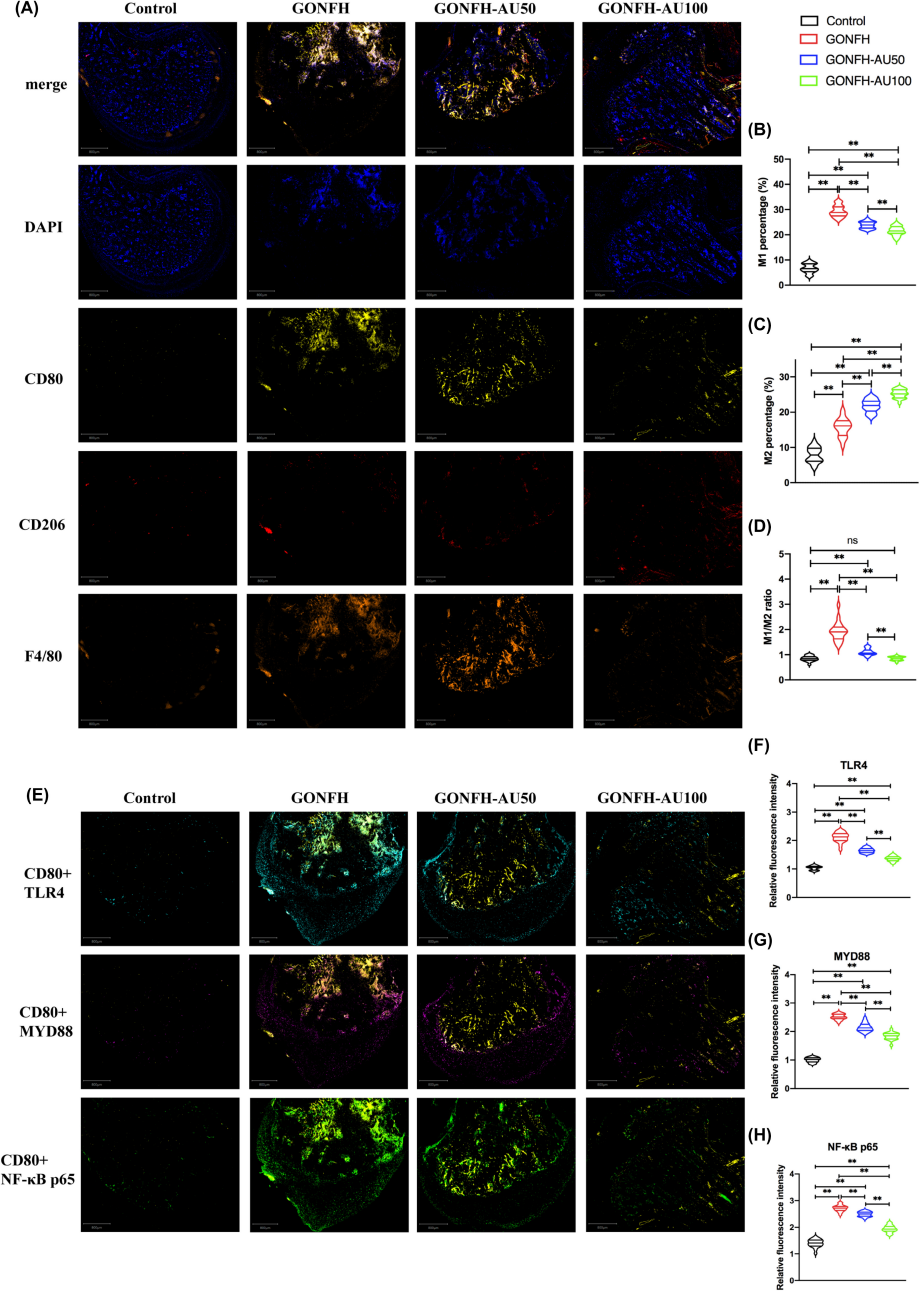
Comparison of macrophage polarization and TLR4/NF‐κB signalling in femoral heads from patients with glucocorticoid‐associated osteonecrosis of femoral head (GONFH) or hip osteoarthritis (HOA). (A) Micrographs of thin sections of femoral heads after multiplex immunostaining. Scale bar, 800 μm. (B–D) Quantitation of proportions of all macrophages that were polarized to the M1 or M2 phenotype in micrographs like those in panel A, B: M1 percentage, C: M2 percentage, D: M1/M2 ratio. Results are shown for 20 animals per condition (**p < 0.01). (E) Micrographs of thin sections of femoral heads after multiplex immunostaining. Scale bar, 800 μm. (F–H) Quantitation of expression of TLR4, MYD44 and NF‐κB p65 in micrographs like those in panel E. Results are shown for 20 animals per condition (*p < 0.05, **p < 0.01).

H&E staining revealed destruction of trabecular bone and marrow structures in the femoral heads of the GONFH group (Figure 3F). Compared with control group, the trabecula is significantly thinner and looser in GONFH group, which showed a larger area of necrosis on the bone with osteocyte lacunae. In contrast with GONFH group, the GONFH‐AU50 group and GONFH‐AU100 group exhibited comparatively preserved trabeculae and fewer osteocyte lacunae. What's more, the trabecula was significantly thicker and tighter and osteocyte lacunae was fewer in GONFH‐AU100 group when compared with GONFH‐AU50 group. These findings suggested that aucubin could ameliorate GONFH in rat model. The bone tissues of the femoral head from GONFH rats contained a significantly increased macrophage M1/M2 ratio and higher levels of TLR4, MYD88 and NF‐κB p65 than those in the control group (Figure 4).

Aucubin partially reversed these effects in a dose‐dependent manner, and these therapeutic effects were associated with a decrease in the overall number of macrophages, decrease in the proportion of M1 macrophages, increase in the proportion of M2 macrophages and downregulation of TLR4, MYD88 and NF‐κB p65 (Figure 4).

### 3.3 Aucubin exerts its therapeutic effects by inhibiting TLR4/NF‐κB signalling and promoting macrophage polarization to the M2 phenotype

**FIGURE 5:**
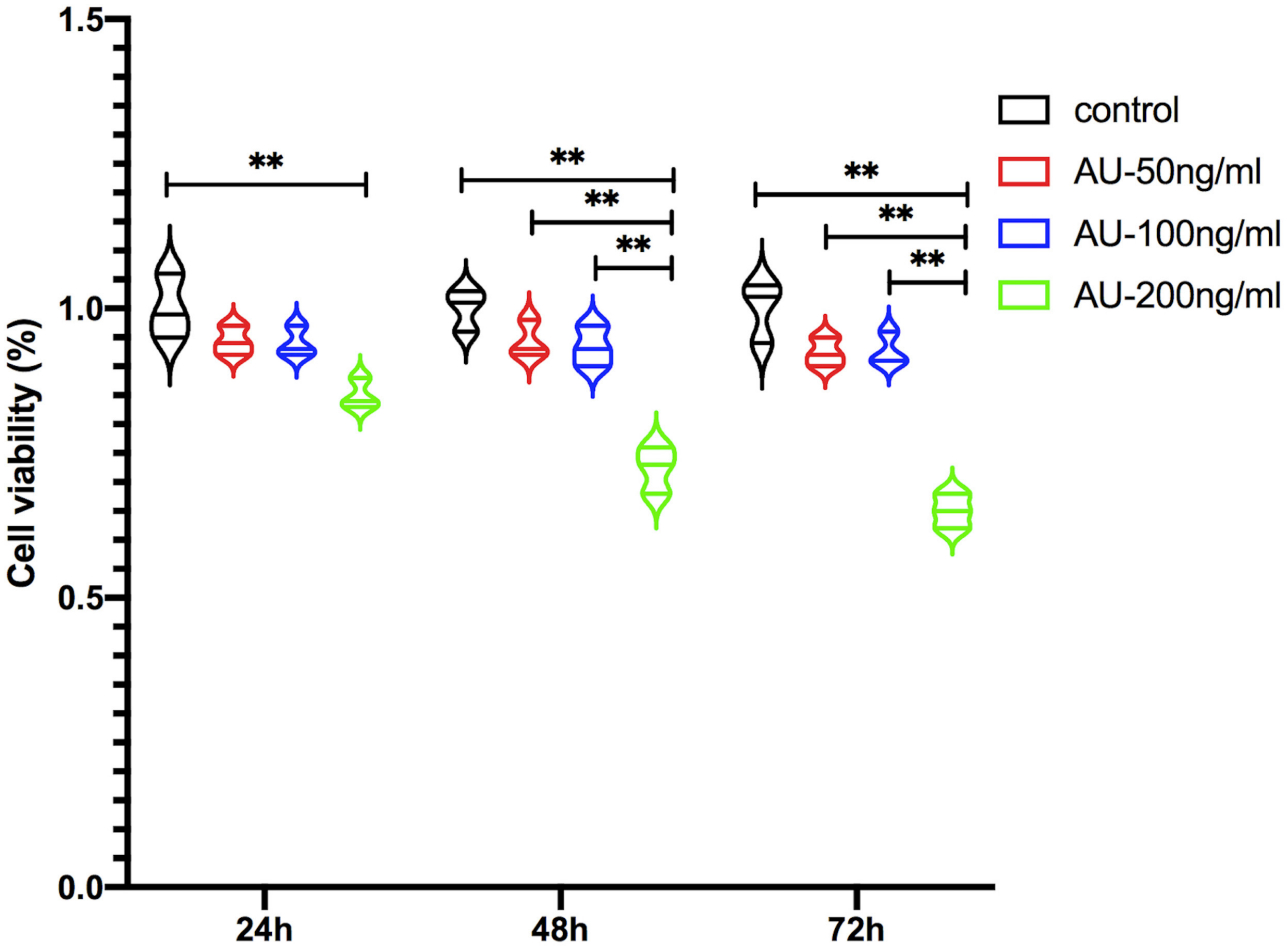
Viability of THP‐1 monocytes that had differentiated into macrophage‐like cells with M1 phenotype and were then incubated for the indicated times with different concentrations of aucubin. Results are shown for three cultures per condition (**p < 0.01).

The experiments in a rat model of GONFH suggested that aucubin dampens TLR4/NF‐κB signalling to shift macrophages from an M1 to M2 phenotype. To test this possibility, we treated a cell culture model of M1 macrophages with aucubin after endogenous expression of TLR4 had been knocked down or not, then we examined TLR4/NF‐κB signalling and M1/M2 phenotype. First, we determined that the cells could be treated with 50 or 100 ng/mL aucubin without significantly reducing their viability, whereas 200 ng/mL of the drug was too high (Figure 5). Since we found the concentration of 100 ng/mL to exert strong therapeutic effects in the rat model of GONFH, we exposed the cell cultures to that concentration of the drug.

**FIGURE 6:**
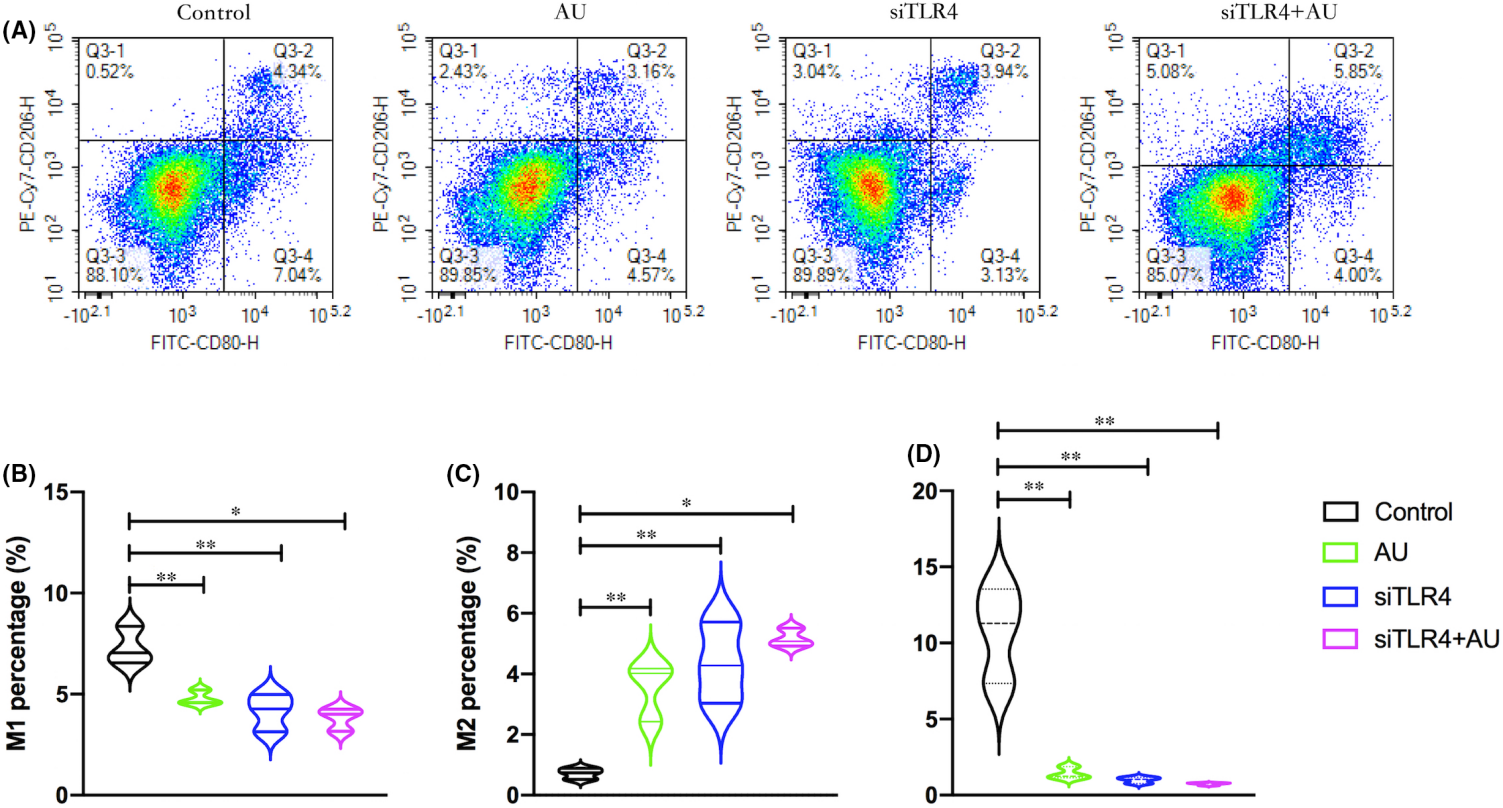
Aucubin shifts polarization of macrophage‐like cells from M1 to M2 phenotype. THP‐1 monocytes expressing normal or knocked‐down levels of TLR4 were treated with aucubin. siTRL4, short interfering RNA targeting TLR4. (A) Flow cytometry to identify populations of M2 macrophage‐like cells (quadrant Q3‐1, CD80^−^ CD206^+^) and M1 macrophage‐like cells (quadrant Q3‐4, CD80^+^ CD206^−^). Results are shown for three cultures per condition. (B–D) Quantitation of proportions of all macrophages that were polarized to the M1 or M2 phenotype. Results are shown for three cultures per condition (*p < 0.05, **p < 0.01).

**FIGURE 7:**
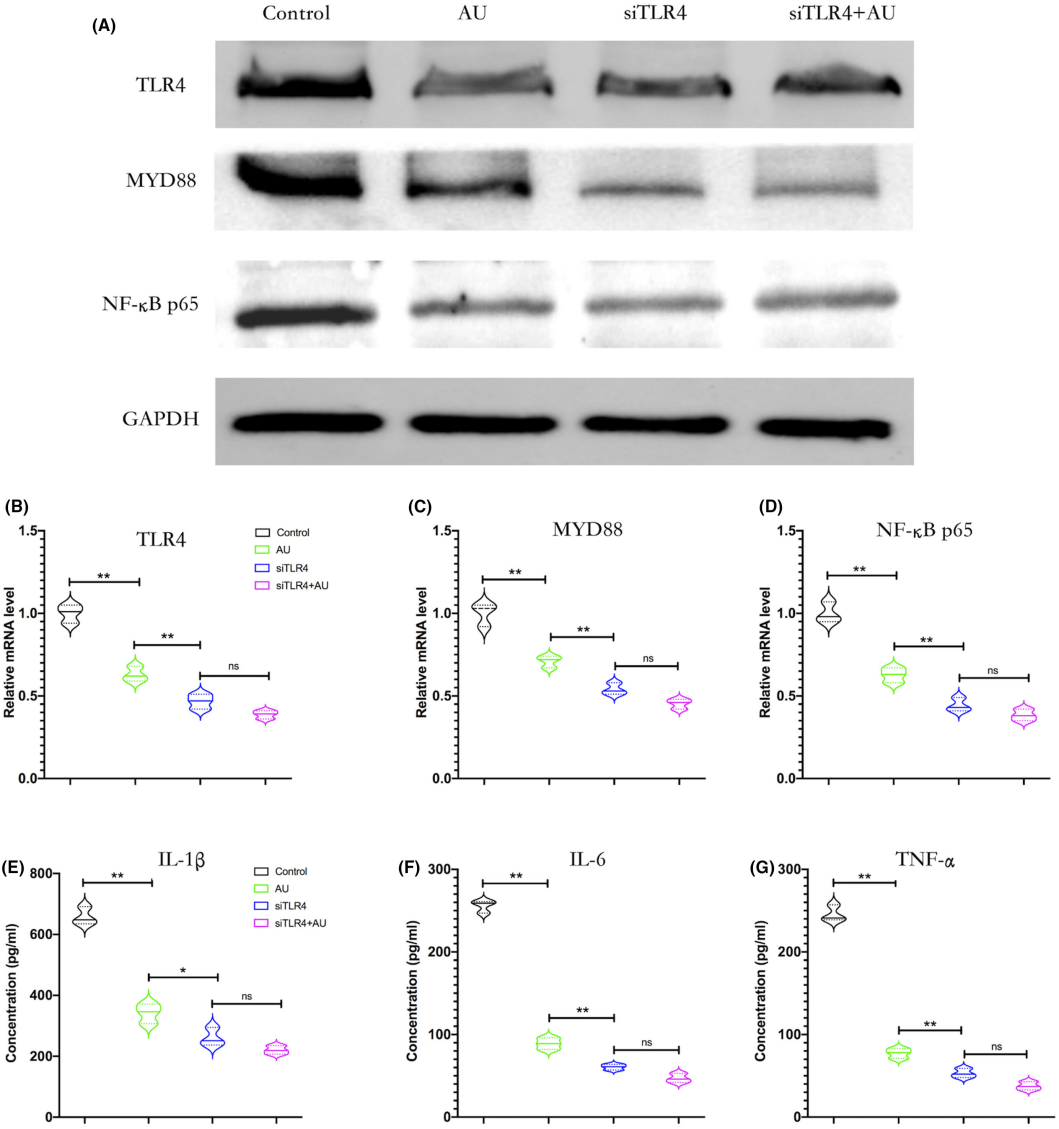
Aucubin inhibits TLR4/NF‐κB signalling to shift macrophage polarization. THP‐1 monocytes expressing normal or knocked‐down levels of TLR4 were treated with aucubin. siTLR4, short interfering RNA targeting TLR4. (A) Western blots of cell lysates after the indicated treatments to detect TLR4, MYD88 and NF‐κB p65 subunit. (B–D) Levels of mRNA encoding TLR4, MYD88, or NF‐κB p65 in cells after the indicated treatments, based on quantitative PCR. (E–G) Levels of IL‐1β, IL‐6 and TNF‐α in the medium of cell cultures after the indicated treatments.

Both aucubin and TLR4 knockdown reduced the proportion of cells that showed an M1‐like phenotype while increasing the proportion that showed an M2‐like phenotype and led to lower ratios of M1 macrophages to M2 macrophages than in control cells (Figure 6). The effects of aucubin in this cell culture model of macrophages involved downregulation of TLR4, MYD88 and NF‐κB p65 at the levels of mRNA and protein, as well as reduced secretion of TNF‐α, IL‐1β and IL‐6 (Figure 7). Knocking down TLR4 on its own exerted similar effects as aucubin, but aucubin did not further enhance the effects of TLR4 knockdown.

## 4 DISCUSSION

Our experiments with patient samples, a rat model and a cell culture model suggest that GONFH involves an increase in the total number of macrophages in the femoral head, particularly macrophages with a pro‐inflammatory M1 phenotype, which is associated with upregulation of TLR4/NF‐κB signalling. The traditional osteopathic phytomedicine aucubin mitigates the pathology of GONFH by dampening this signalling and simultaneously reducing the proportion of M1 macrophages and boosting the proportion of anti‐inflammatory M2 macrophages. This translates to overall lower secretion of the pro‐inflammatory cytokines TNF‐α, IL‐1β and IL‐6 by macrophages. These findings rationalise the use of aucubin against GONFH and identify TLR4/NF‐κB signalling and macrophage polarization as druggable targets in the disorder.

Our work is consistent with previously established links between macrophages and pathogenesis of GONFH,^18^ particularly observations from our group^9^ and others^8^ that the disorder involves enrichment of macrophages, especially M1 macrophages, in the femoral head. An excess of M1 macrophages relative to M2 macrophages likely helps drive the chronic inflammation characteristic of GONFH.^8^ Inhibiting the polarization of M1 macrophages and promoting the polarization of M2 macrophages would impede the progression of GONFH and facilitate the reparation of GONFH. Our work is also consistent with previous reports that TLR4/NF‐κB signalling regulates macrophage polarization and is dysregulated in GONFH.^26^ In fact, such TLR4‐mediated dysregulation of macrophage polarization has been described in liver cancer, focal cerebral ischemia–reperfusion injury and spinal cord injury.^23^, ^24^, ^27^ Damage‐associated molecular patterns are released from necrotic bone, activating TLR4 to induce pro‐inflammatory responses by macrophages^26^ involving activation of pathways mediated by MYD88 and NF‐κB.^28^, ^29^ In osteonecrosis of the femoral head induced by steroids or corticosteroids, NF‐κB activation helps increase the proportion of M1 macrophages,^8^ and supressing this activation can mitigate the disease,^29^ which indeed appears to be part of aucubin's therapeutic mechanism, as we have shown here and as others have shown in previous work.^17^ Hence, we propose that modulating macrophage polarization via the TLR4/NF‐κB signalling pathway could represent a novel therapeutic target for GONFH.

Aucubin is the most widespread iridoid glycoside in plants, including *Cornaceae, Garryaceae, Orobanchaceae, Globulariaceae, Eucommiaceae, Scrophulariaceae, Plantaginaceae and Rubiaceae*.^30^ Aucubin is a glycoside whose aglycone (i.e., aucubigenin) binds to the glucose group using an O‐glycosidic bond.^31^ Aucubin only exhibits biological activities when the glycoside is converted into its aglycone form through deglycosylation, either in vivo or in vitro. In vivo pharmacokinetic testing proves the bioavailability of aucubin to be higher with the intraperitoneal route (76.8%) than the oral route (19.8%).^31^ This may result from the unstable nature of aucubin in the acidic gastric juice, poor absorption onto the gastrointestinal tract due to low lipophilicity and possible first‐pass effects in the liver.^32^ Aucubin shows promise for a variety of therapeutic and biomedical applications because of its various biological activities.^31^ It was reported that aucubin could inhibit osteoclast activity, promote osteoblast activity, enhance bone formation and promote angiogenesis reduce bone resorption.^33^, ^34^, ^35^

In clinical practice for treating GONFH, aucubin's producing plants are used as components of Chinese medicine formulae, like Duhuo Jisheng Decoction.^36^ Chinese medicine formulae could be used as the major treatment or adjuvant therapy of other treatments, such as tantalum rod implantation, core decompression. Except for those patients need total hip replacement, all the patients could receive Chinese medicine formulae immediately after GONFH is diagnosed if there is no contraindication. The present study confirms and extends previous findings that aucubin can mitigate bone loss and apoptosis due to osteoporosis, osteoarthritis,^33^, ^37^ as well as inhibit osteoclast activity, enhance bone formation and density, thicken cortical bone and tighten trabecular bone in osteoporosis models.^33^ Aucubin has been shown to promote VEGFR2‐mediated angiogenesis,^38^ which can promote bone repair and regeneration in osteonecrosis and other bone disorders.^39^ We have also shown that aucubin inhibits apoptosis and promotes autophagy in osteoblasts.^7^ In this study, we found that aucubin could ameliorate the bone necrosis, bone mineral loss, destruction of trabecular bone and marrow structures in rat model of GONFH. Our result indicated that aucubin could prevent bone loss after osteonecrosis, although it can't suppress the occurrence of GONFH. However, few previous study provided evidence about whether aucubin treated GONFH through promoting angiogenesis and bone formation and/or through inhibiting bone resorption. In future studies, it's necessary to explore the detail treatment mechanism of aucubin on GONFH. Additionally, our research revealed that aucubin could reduce the percentage of M1 macrophages and decrease the secretion of TNF‐α, IL‐1β and IL‐6 from macrophages in vitro. Considering the interplay between the TLR‐4/NF‐κB signalling pathway, macrophage polarization and GONFH, we propose that aucubin may mitigate GONFH by modulating macrophage polarization through the TLR‐4/NF‐κB signalling pathway. This should be investigated in future work as another potential mechanism behind the phytocompound's therapeutic effects against GONFH.

There are several limitations in our study. First, we only applied knocking‐down TLR4 to test aucubin's therapeutic potential in suppressing TLR4/NF‐κB signalling. If a group with over‐expression of TLR4 was added, the evidence of aucubin's therapeutic potential in suppressing TLR4/NF‐κB signalling would be more convincing. Second, our study focused on the effects of aucubin on regulating macrophage polarization and TLR4/NF‐κB signalling in GONFH. While the mechanism of aucubin on preventing bone loss, promoting revascularization and restoration of haematopoietic bone marrow after GONFH is still unknown, and its effects on osteoblasts and osteoclasts, bone marrow haematopoietic stem cell in GONFH need further studies to examine. Third, in animal experiment, we only chose 8 weeks as observation time. It would provide more evidences to observe the effects of aucubin on treating GONFH, as well as regulating macrophage polarization and TLR4/NF‐κB signalling, if there were more observation times, such as 4, 8 and 12 weeks.

## 5 CONCLUSIONS

Our results suggest that GONFH involves activation of TLR4/NF‐κB signalling, which shifts macrophage polarization from an M2 to M1 phenotype in the femoral head. These phenomena are reversed by the traditional orthopaedic medicine aucubin, which thereby mitigates the pathology of GONFH. Our findings justify the continuing use of aucubin against GONFH and emphasise the usefulness of targeting macrophage polarization for treating the disorder.

## AUTHOR CONTRIBUTIONS

**Chen Yue:** Conceptualization (lead); funding acquisition (lead); project administration (lead); writing – review and editing (equal). **Guofeng Cui:** Conceptualization (equal); funding acquisition (equal); methodology (equal). **Yan Cheng:** Data curation (equal); investigation (equal); methodology (equal). **Xue Zhang:** Investigation (equal); methodology (equal); resources (equal); software (lead). **Hong‐feng Sheng:** Supervision (equal); validation (equal); visualization (equal). **Yidan Yang:** Validation (equal); visualization (equal); writing – original draft (equal). **Jiayi Guo:** Data curation (equal); formal analysis (equal); methodology (equal); resources (equal). **Youwen Liu:** Data curation (equal); formal analysis (equal); investigation (equal). **Bin Xu:** Conceptualization (equal); funding acquisition (equal); methodology (equal); writing – original draft (equal); writing – review and editing (equal).

## FUNDING INFORMATION

This work is supported by National Natural Science Foundation of China (82074472); The Zhejiang Provincial Natural Science Foundation (LQ22H060003); Key Science and Technology Research Projects in Henan Province (242102310518 and 222102310368).

## CONFLICT OF INTEREST STATEMENT

The authors have no relevant financial or non‐financial interests to disclose.

## CONSENT

The patients consented to the femoral heads samples being taken for the purpose of research and also consented to their publication.

## Supporting information


Table S1.

## Data Availability

The datasets used and/or analysed during the current study are available from the corresponding author on reasonable request.
