# Supplementary material for: Aucubin suppresses TLR4/NF‐κB signalling to shift macrophages toward M2 phenotype in glucocorticoid‐associated osteonecrosis of the femoral head
Source: J Cell Mol Med. 2024 Aug 9;28(15):e18583. doi: 10.1111/jcmm.18583 (PMC11315675; doi:10.1111/jcmm.18583)
Supplement: Supplementary file 1 — Table S1. [file JCMM-28-e18583-s001.doc]

Supplementary Table S1. Real-Time PCR Primers

| Gene | Forward primer | Reverse primer |
| --- | --- | --- |
| TLR4 | 5’-CGTGCAGGTGGTTCCTAACA-3’ | 5’-GTGGGGATGTTGTCAGGGATT-3’ |
| MYD88 | 5’-TGGCCTTGTTAGACCGTGA-3’ | 5’-TGGCCTTGTTAGACCGTGA-3’ |
| NF-κB p65 | 5’-CTGGGCACCAGTTCGATGG-3’ | 5’-GACAGCATAAGGCACACACTT-3’ |
| GAPDH | 5’-GACCAGGTTGTGTCCTGTGA-3’ | 5’-AGCTTGACGAAGTGGTCGTT-5’ |
